# Impedance-Based Biosensing of *Pseudomonas putida* via Solution Blow Spun PLA: MWCNT Composite Nanofibers

**DOI:** 10.3390/mi10120876

**Published:** 2019-12-13

**Authors:** Craig Miller, Madison Stiglich, Mark Livingstone, Jordon Gilmore

**Affiliations:** Department of Bioengineering, Clemson University, Clemson, SC 29634, USA; clm6@g.clemson.edu (C.M.); mstigli@g.clemson.edu (M.S.); mblivin@g.clemson.edu (M.L.)

**Keywords:** solution blow spinning, phase angle, magnitude, impedance

## Abstract

Quantifiable sensing of common microbes in chronic wounds has the potential to enable an objective assessment of wound healing for diagnostic applications. Sensing platforms should be robust, simple, and flexible to provide clinicians with a point-of-care tool. In this work, solution blow spun poly (lactic acid)/multiwalled carbon nanotube nanofiber composites are used to detect the presence and concentration of *Pseudomonas putida* in vitro using changes in impedance. Impedance microbiology (IM) is a well-documented diagnostic technique used in many applications, including cancer detection, tuberculosis screening and pregnancy tests. Twenty-four hour real-time measurements of the equivalent circuit of three culture media were taken with an inductance, capacitance, and resistance (LCR) meter. Variations in impedance were calculated to correspond to the growth of *P*. *putida.* Additionally, instantaneous measurements of bacterial cultures were taken over a one-minute time point to display the fast sensing of bacterial load via IM. This proof-of-concept shows that conductive solution blow spun fiber mats is a valid fabrication technique to develop in situ wound dressing impedance sensors. Study results indicate successful measurement and quantification of bacterial growth in this proof-of-concept study.

## 1. Introduction

Chronic wounds are often sites of excessive bacterial colonization, leading to infection, delayed healing, amputation, and/or mortality [1]. *Staphylococcus aureus*, *Enterococcus faecalis* and *Pseudomonas aeruginosa* are common pathogens found in non-healing wounds, where concentrations above 10^5^ colony forming units (CFU)/gram (g) have the potential to impede the healing process [2] Commonly, bacteria seek to invade the epidermis layer and excrete toxins and virulence factors into the bloodstream. Most of these organisms produce biofilms, a community of bacteria highly resistant to common treatment therapies. Biofilms become resistant through a phenomenon called quorum sensing. This communication system modifies gene expression via cell-to-cell communication among neighboring bacteria cells. These sensing systems are regulated by a set of molecules called autoinducers (AIs) or autoinducer peptides (AIPs) whose concentration is a function of cell population density [2,3]. Bioluminescence, virulence factors, sporulation, and biofilm formation depend on the upregulation of AIs [4]. This requires clinicians to find ways to prevent the onset of infection [5]. *Psuedomonas putida (P. putida)* is a part of the pseudomonas fluorescens species and is a Gram-negative, rod-shaped bacterium [5]. Under ultraviolet light, it fluoresces a yellow green dye called pyoverdine [5,6]. *P. putida* prefers a moist environment; wounds with high exudate levels provide the environment for colonization [5]. This microorganism is a common nosocomial agent found on medical devices, implants, and mucous membranes of patients [5]. Hence, amputees, or patients with stents, pacemaker, etc. are at a higher risk. In the past, *P. putida* was thought to be nonpathogenic in Homo Sapiens, but it has been recognized as a cause for true infection over the past few decades [5]. In this study, *P. putida* is used to test analyze the use of solution blow spun nanofiber mats as a transducer for impedance sensing. 

**Figure 1:**
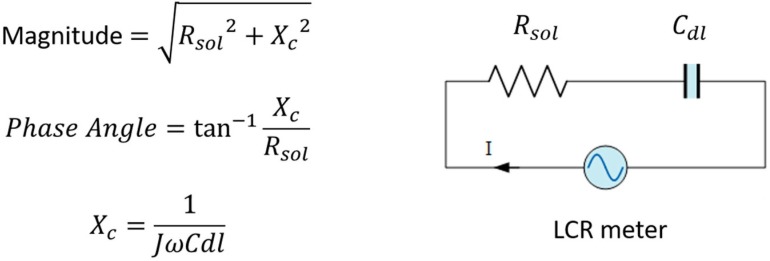
Schematic diagram of impedance microbiology theory.

There is a need to quantify wound infection status and fabrication of technology that would enable in situ detection of bacterial load. Early detection would allow clinicians to treat infections in its earlier stages and avoid antimicrobial resistance to empirical antibiotics [7]. Impedance based technology provides quantifiable information by the application of an alternating input signal and measurement of the system responding magnitude and phase angle. Previously, bioimpedance has been deployed to assess wound status in human and animal subjects [8,9]. Monitoring of cell number, viability and metabolic activity have all been monitored via impedance response [10,11,12]. Impedance microbiology is a technique that correlates resistance and reactance values to biological phenomena. Resistance is proportional to the ionic concentration of the extracellular fluid at the electrode surface, while reactance correlates to cell mass and is a good indicator of cell proliferation at the electrode surface [10,11,12]. For this study we define the complex impedance is expressed in polar form as seen in Equation (1).

(1) $$Complex\;Impedance\;\left(Z\right)=\left|Z\right|e^{j\Theta }$$

where |Z| is the magnitude and Θ is the phase angle of the complex impedance. Figure 1 gives a mathematical representation of impedance and the equivalent circuit of the sensing area, where *C_dl_* is the double layer capacitance that is determined by the capacitance of the cell membrane. *R_sol_* is the solution resistance. Thus, impedance biosensors could quantify cell proliferation by calculating the equivalent impedance of the wound environment. Wearable sensor modalities should be flexible, adequately conform to wound geometry, and limit shear stress induced at the wound surface.

In this study, we examined the use of impedance to detect the presence and concentration of *P. putida* in vitro via a label-free, antibody-free method using poly-l-lactide (PLA): multi-walled carbon nanotube-solution blow spun-printed circuit board (PLA: MWCNT-SBS-PCB) transducers. The frequency used for all measurements was 200 Hz and kept at a constant value during testing cycles. Twenty-four-hour real-time impedance measurements were taken on bacteria culture with an initial concentration ranging from 10^3^–10^7^ CFU/mL to examine any changes in impedance values. A calibration curve was constructed to display bacterial concentration as a function of the change in the magnitude and phase angle values. Instantaneous testing was done on culture media to examine the feasibility of rapid bacteria detection with PLA: MWCNT-SBS-PCB transducers. PLA: MWCNT-SBS-PCB transducers were able to rapidly quantify planktonic bacterial load through the measurement of impedance magnitude and phase angle over a one-minute testing cycle. Impedance-based bacterial sensing has been utilized in the past to identify and quantify bacterial load [13,14,15,16,17,18,19]. However, none has included the use of conductive nonwoven nanofibers as the main transduction component, which have the potential for customizable shape and size for in situ incorporation. Other studies utilize fabrication techniques that would render the use of impedance sensing in a clinical setting too expensive, such as photolithography. PLA: MWCNT-SBS-PCB transducers are less expensive than such methods and are produced in significantly less time. This fabrication technique could be incorporated into a wound dressing or on a wearable sensor substrate by solution blow spinning a layer onto the substrate directly. The fibers act as the transducer themselves due to the porosity of the fiber mat which enables the detection of the impedance change as bacteria and other cells interact and alter the fiber orientation.

## 2. Materials and Methods 

### 2.1. Bacterial Cultures

Cell culture media was composed of lysogeny broth (liquid media) and agar (solid media) (Fisher Scientific, St. Louis, MO, USA) in deionized water. Media was sterilized at 120 °C for 1 h via autoclave. For liquid media experiments, *P. putida* was inoculated (P. putida) in 3 mL of 2% LB media. Bacteria were cultured on an orbital shaker for 24 h at 37 °C. In order to normalize data over three samples, a calibration curve was formed to relate concentration levels to optical density values (OD). OD values were measured via a Nano Drop 600 (Thermo Scientific, Wilmington, DE, USA) UV-vis system. Concentrations of 103,105, and 107 CFU/mL were tested and their respective starting OD values were kept constant before the start of each test cycle.

### 2.2. Nanofiber Sensor Fabrication and Multi-Walled Carbon Nanotube-Solution Blow Spun-Printed Circuit Board (MWCNT-SBS-PCB) Board Characterization

PLA pellets (Mn = 75,000 g/mol) were purchased from Jam Plast Inc. (Ellisville, MO, USA). Chloroform (CHCl 3 CAS 67-66-3) and MWCNT (CAS308068-56-6) were purchased from Sigma-Aldrich (St. Louis, MO, USA). Fiber mats were fabricated via solution blow spinning conductive solutions onto 10 cm × 25 cm printed circuit boards (PCBs). 

**Figure 2:**
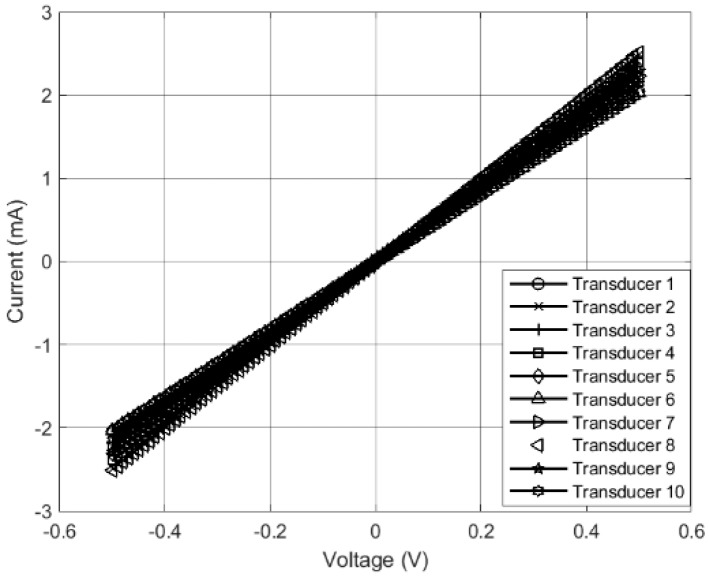
Current-voltage curve of ten solution blow spun-printed circuit board (SBS-PCB) transducers.

Fibers were generated using a 115 mL/h flow rate at a pressure of 30 psi. In order to use MWCNT-SBS-printed circuit boards (PCBs) as the main transducer in an impedance study, linearity between current and voltage is important. A custom two-point probe system was used to measure the I-V curves of ten different transducers. The applied voltage ranged from −500 mV to 500 mV and the induced current was measured. The average resistance of the PCBs was 220 ± 16 Ω (n = 5). As shown in Figure 2, I-V curves of all ten boards showed a linear dependence between the applied voltage and the measured current. Given the importance of an accurate initial resistance, consistently generating large numbers of fiber mat-coated boards proved to be difficult. Out of 50 boards, roughly 30 fell into the above range. The 20 boards falling out of the desired resistance range were not used in the testing phase. In order to test the use of MWCNT-SBS-PCBs as the main transducer, only fiber mats falling in the above range were used to start testing. Fiber mats used in this study were further characterized in a previous study [20].

**Figure 3:**
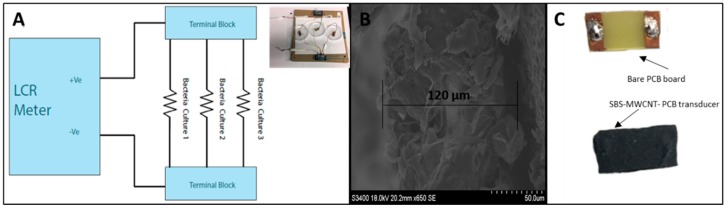
(**A**) Equivalent circuit of Impedance Test Circuit. (**B**) Cross-sectional view SBS nanofiber mat (diameter = 120 µm). (**C**) Front view of PCB board and SBS-PCB transducer.

Figure 3 shows a graphical representation of the test setup used to continuously detect the presence of bacteria in vitro. A parallel connection between three Petri dishes was made in order to test multiple samples under the same condition. 10 × 25 cm MWCNT-SBS-PCB transducers were placed flat in the Petri dishes and a connection was made through the top of the Petri dishes, on the backside of the boards to the inductor, capacitance, and resistance (LCR) meter. This setup provided consistent impedance sensing results with minimal drift current, while maintaining conditions commonly used to culture microbial and mammalian cells. For instantaneous testing, one transducer was placed in a beaker and 2 mL of bacterial culture was added to assess concentration levels. Data from the instantaneous test was graphed vs. its starting concentration. Impedance of the bacterial pathogen cultured on Petri dishes was measured using a 1 V sine wave at 200 Hz; 200 Hz was found to be the most sensitive and gave a greater change in impedance than higher frequencies. Other work also supports this observation, as it was shown by Yang et al. that bacterial cultures behave like low-pass filters [21]. Re-calibration of the LCR meter and new fiber mat transducers were used in each testing cycle. All measurements were performed at room temperature under a custom-made enclosure to ensure minimal media evaporation. Cycle duration was set to 10 points per minute to allow continuous 24-h monitoring. The 24-h test is a simulation of a real-time point-of-care (POC) diagnostic approach that could be used to monitor wound progression at home. Instantaneous, one-minute testing cycle was done using one transducer in a beaker with 2 mL of culture media to assess the response time of transducers and could be later used to quickly assess the wound state in a clinical POC setting.

## 3. Results

### 3.1. 24-Hour Continuous Impedance Sensing

**Figure 4:**
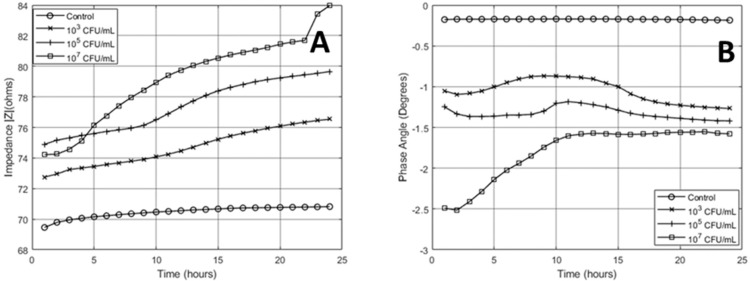
(**A**) Twenty-four-hour impedance phase angle. (**B**) Twenty-four-hour impedance magnitude.

Transducers falling in the range of 220 +/− 15% Ω were used in all sensing conditions. The average starting impedance for the parallel setup before testing was 73.7 Ω with phase angle −0.0228° ± 2.93 Ω at 0.0072°. The sensing cycle for the 24-h measurement began 30 s after inoculation of pre-determined concentration. Figure 4 shows the impedance values of the experimental groups. The measured values were averaged out over each hour. The highest concentration tested (107 CFU/mL) had the highest corresponding impedance magnitude and the lowest phase angle value after 24 h of testing. This is an indication of higher cell density in the local testing area. The effect of bacterial growth on transducers dominated the magnitude response over the testing cycle. A small amount of the change in magnitude response can be attributed to media evaporation (across all groups). The variation in the control was 5% and treatment groups varied by 16%, 20%, and 41% for the 103, 105, and 107 concentrations, respectively. The magnitude in the 107 group was lower than the 105 group, but increased to a higher value after five hours. This response could be due to lower media conductivity caused by planktonic/mobile bacteria in the lag phase. Over the 24-h testing cycle, magnitude values for all treatment groups increased, which is an indication of log phase bacterial growth. There was also a change in the slope in all treatment groups where the point of change occurred earlier for higher concentrations.

There was a significant difference in the phase angle values between all groups within the first hour of testing (n = 3, *p* < 5%). Bacterial cells can be modelled as capacitors as previous models of bacteria have been reported [22,23]. Higher concentrations gave rise to a more negative phase angle value in all treatment groups. Values for all treatment groups increased over the first five hours. After this gradual increase, there is a shift in te response where phase angle values start to stabilize and decrease slightly. Again, this change occurs at an earlier time for higher concentrations. This response is due to bacterial cell adhesion to the electrode surface. As cells continue to grow and populate the testing area, the cell membrane and biofilm orientation give rise to a decreasing phase angle. Magnitude and phase values can be used as a dual validation. This is where impedance can be exploited. This would allow quantification of bacterial concentration load based on two parameters as opposed to only one.

**Figure 5:**
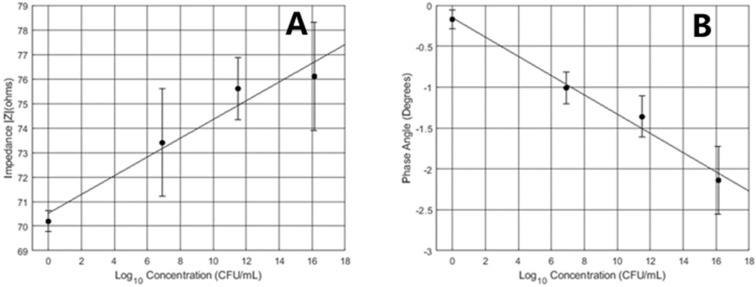
(**A**) Twenty-four-hour impedance phase angle. (**B**) Twenty-four-hour impedance magnitude.

From the 24-h test, a standard curve was generated as can be seen in Figure 5. Data points were averaged for each concentration up to 10 h. The calibration curve displayed a positive correlation between the impedance magnitudes and the logarithmic bacterial concentration with an R-squared value of 0.96. The phase angle standard curve had the opposite response with an R-squared value of 0.98.

### 3.2. Instantaneous Metabolic Sensing

**Figure 6:**
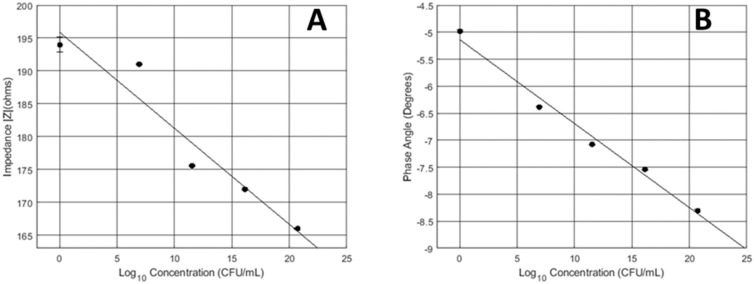
Calibration curve. Using MWCNT-SBS-PCB transducers to instantaneously detect bacterial concentration. (**A**) Magnitude. (**B**) Phase angle.

Further work was done to assess this method in an instantaneous fashion. For chronic wound applications it is worth noting that in any situation, there will always be a population of mature, multi-species bacteria. Instantaneous testing was done on the same concentrations as the above procedure where 2 mL of culture media was used. This method was used to assess the determination of bacterial concentration with MWCNT-SBS-PCB transducers. Impedance magnitude response over the one-minute testing cycle had a negative correlation with bacterial concentration. This response is due to the dispersion of planktonic cells in the culture media. Therefore, the media is more conductive with more bacteria. As a result, magnitude values decrease. Phase angle values responded similarly to the continuous testing. The slope from the instantaneous test was −0.36 and −3.4 for the phase and magnitude, respectively. The slope for the magnitude and phase from Figure 5 was 0.89 and −0.27, respectively. This shows that the instantaneous testing was more sensitive than the continuous cycle. This could be due to faster ionic movement in the liquid phase of the instantaneous test setup as compared to the solid media. Based on the slope of the equation from the standard curves of Figure 6 both the magnitude and phase was more sensitive for the instantaneous test. This test shows that bacteria respond like capacitors in the planktonic growth phase and in the biofilm growth phase. Future studies are needed to assess MWCNT-SBS-PCB instantaneous impedance response to biofilm cultures.

## 4. Conclusions

This work examines the use of a conductive coating to detect the presence and growth of bacteria in vitro. Impedance spectroscopy was used to detect the physical interaction of the coating with the culture media and bacteria population. For the first time, solution blow spinning has been used to generate conductive fiber transducers capable of detecting the presence and concentration of bacteria in a LB media culture. This technology has the potential to provide rapid, real-time quantitative data about the bacterial activity in a culture media. Additionally, this method provides an economical method to generate biosensors capable of detecting infection. Continuous monitoring (24-h testing) provided a way to simulate bacterial growth in a culture environment over a longer period, and may have applications in long term, remote patient monitoring, and telehealth. Long-term monitoring allows for the time-wise characterization of the change in impedance as bacterial colonization and biofilm formation begin to surround transducers. Instantaneous measurements (one-minute testing) were able to detect the presence of bacteria within seconds and would be useful in point-of-care clinical applications where immediate quantification is important for diagnosis. Conductive fiber mats will be used as a coating in future biosensor construct fabrication. Selective sensing will be developed by functionalizing the coating surface with bio-recognition elements (i.e., antibodies, enzymes, or signaling molecules). Impedance spectra provides rapid, quantifiable information about bacterial growth and could be used as a valid analytical and diagnostic tool in wound care clinical settings. Further work will be done to increase the sensitivity of continuous impedance sensing with SBS nanofibermats. Furthermore, improvements must be made in order to accurately mass-produce transducers with a defined electrode pattern.
